# All-cause and cause-specific mortality associated with diabetes in prevalent hemodialysis patients

**DOI:** 10.1186/1471-2369-13-130

**Published:** 2012-10-01

**Authors:** Abdus Sattar, Christos Argyropoulos, Lisa Weissfeld, Nizar Younas, Linda Fried, John A Kellum, Mark Unruh

**Affiliations:** 1 Department of Epidemiology and Biostatistics, Case Western Reserve University, Cleveland, USA; 2 Renal and Electrolyte Division, University of Pittsburgh, Pittsburgh, USA; 3 Department of Biostatistics, University of Pittsburgh, Pittsburgh, USA; 4 Department of Critical Care Medicine, University of Pittsburgh, Pittsburgh, USA; 5 Nephrology Division, University of New Mexico, Albuquerque, USA

**Keywords:** Cox regression, Diabetes, ESRD, Hemodialysis, Proportional hazard, Survival model, Time varying coefficient model, Time dependent covariate.

## Abstract

**Background:**

Diabetes is the most common risk factor for end-stage renal disease (ESRD) and has been associated with increased risk of death. In order to better understand the influence of diabetes on outcomes in hemodialysis, we examine the risk of death of diabetic participants in the HEMODIALYSIS (HEMO) study.

**Methods:**

In the HEMO study, 823 (44.6%) participants were classified as diabetic. Using the Schoenfeld residual test, we found that diabetes violated the proportional hazards assumption. Based on this result, we fit two non-proportional hazard models: Cox’s time varying covariate model (Cox-TVC) that allows the hazard for diabetes to change linearly with time and Gray’s time-varying coefficient model.

**Results:**

Using the Cox-TVC, the hazard ratio (HR) for diabetes increased with each year of follow up (p = 0.02) for all cause mortality. Using Gray’s model, the HR for diabetes ranged from 1.41 to 2.21 (p <0.01). The HR for diabetes using Gray’s model exhibited a different pattern, being relatively stable at 1.5 for the first 3 years in the study and increasing afterwards.

**Conclusion:**

Risk of death associated with diabetes in ESRD increases over time and suggests that an increasing risk of death among diabetes may be underappreciated when using conventional survival models.

## Background

Diabetes is one of the leading causes of end stage renal disease in the U.S. [1] and ESRD due to type 2 diabetes mellitus has been increasing worldwide [2,3]. Diabetes has known macrovascular complications such as myocardial infarction and stroke. In addition, diabetes affects the microvascular circulation, impairs neutrophil function which increases the risk of infections which may be life threatening [4,5], and also leads to an accumulation of advanced-glycosylation end products and oxidative stress. Diabetic status has been associated with increased cause of death in international dialysis cohorts [1,6]. In the case of diabetes, effects of the disease may accumulate over time resulting in a hazard function that is increasing at a greater rate over time than that in subjects without diabetes. Since diabetes is such a prevalent and important comorbid condition in the ESRD population, it is critical to better understand the longitudinal association of diabetes with cause-specific and overall mortality. The relationship between diabetes and time-to-death in ESRD populations is usually demonstrated using the Cox proportional hazards (Cox-PH) model. Though the Cox-PH model is a useful tool for assessing the relationship of diabetes to time-to-event data, it requires that the proportional hazards (PH) assumption be met; specifically that the hazard in the diabetic group is a constant proportion of the hazard in the non-diabetic group. Cox-PH model may not perform well when used to study exposures that are cumulative over time [7].

There were a substantial number of diabetics studied in the HEMO Study, a large multi-center trial of hemodialysis patients which examined the effects on hemodialysis patient survival of dose (eKt/V 1.45 vs. 1.05) and hemodialyzer flux (high vs. low). The HEMO Study provided an opportunity to assess the extent to which diabetes influences survival in prevalent hemodialysis patients since the study has a prospective follow-up, careful characterization of the severity of comorbid conditions, rigorous classification of the cause of death, and used diabetes as a stratification variable. Since dialysis dose and membrane flux were interventions in the HEMO Study, stratification by diabetes ensures that these factors were accounted for when assessing the relationship between diabetes and survival outcomes. In this report, we take advantage of the design features of the randomized controlled clinical trial to examine the risk of death for the diabetic participants in the HEMO study and to compare different approaches for estimation of the risk of death in diabetics with ESRD. To address the issue of non-proportional hazards we applied models that allow for non-proportionality of conditional hazards through the introduction of spline-based models. Gray’s spline-based extension of the Cox proportional hazards model allows for modeling of the effects of covariates without making the assumption of proportional hazards [8,9]. Specifically, we examine the risk of all-cause and cause-specific mortality of diabetic patients on hemodialysis over time and compare the performance of the standard Cox Model, a Cox model assuming a linear change in the hazard with time (Cox-TVC) and Gray’s model in providing an estimate of risk of death in this patient population.

## Methods

### Subjects

The HEMO Study was a fifteen-center randomized clinical trial of the effects of hemodialysis dose and membrane flux on mortality and morbidity in patients undergoing chronic dialysis [1]. Patients in this study were randomized to either standard or high dose (eKt/V of 1.05 vs. 1.45, respectively) and to either high or low flux membranes (beta-2 microglobulin clearance of <10 ml/min or >20 ml/min, respectively). Enrollment in the HEMO Study began in March 1995 and ended in October 2000 with a total of 1846 patients enrolled in the study. Patient eligibility criteria have been described previously [6]. The primary endpoint was all-cause mortality [10]. The Institutional Review Boards at the 15 institutions approved the study protocol and written informed consent was obtained from all study participants.

### Data collection

Demographic information and clinical history were collected through review of medical records and self-reported questionnaires. The race of the respondent was assessed by self-report and categorized as African-American or non-African American include those self-identified as white, Asian, Native American or ‘other’. Clinical data including laboratory measurements were obtained using standardized protocols. Comorbidity was assessed at baseline using the Index of Coexistent Disease (ICED) calculated without using the diabetes score [6,11,12]. The ICED aggregates the presence and severity of 19 medical conditions and 11 physical impairments into 2 summary indices: the Index of Disease Severity (IDS) and the Index of Physical Impairment (IPI) [12]. Diabetes was defined using the ICED classification as present if the patient had been previously diagnosed with diabetes, had been prescribed a diabetic diet, was receiving insulin or oral hypoglycemic agents or had been previously admitted to the hospital for hyperglycemia or ketoacidosis.

### Classification of cause-specific mortality in HEMO

The duration of follow-up monitoring ranged from 0.003 to 6.639 yr, depending on the time of randomization. The mean actual follow-up duration was 2.84 yr. The determination of cause-specific mortality in the HEMO Study has been previously described [6,7,9]. Briefly, causes of deaths were independently reviewed by two clinicians blinded to the intervention assignments. The members assigned one primary and up to three secondary causes of death based on their clinical judgment from the narrative description of the death, hospital discharges, laboratories, and study data. When agreement could not be reached, the case was adjudicated by the full Outcome Review Committee. The cause-specific infectious, cardiac, and cardiovascular deaths were selected since these are the most common etiologies of death among hemodialysis patients. The four cardiac causes of death were: (*1*) Ischemic Heart Disease; (*2*) congestive heart failure; (*3*) arrhythmias, and (*4*) other heart diseases. The categorization of cardiovascular deaths included cardiac, cerebrovascular and vascular causes of death.

### Covariates for all-cause and cause-specific mortality

In order to account for residual confounding on mortality we adjusted our survival analyses for the following baseline patient characteristics that have been found to be associated with the mortality of ESRD patients in previous studies. 1) *Demographic:* age, gender, race and body mass index (BMI) *2) Dialysis Related Factors:* Duration of ESRD (also known as “vintage”), residual renal function (absent or present based on a residual urine output of less than 200 ml/day) and dialysis access. Dialysis access was defined as dialysis catheter vs. all other (Arteriovenous Fistula (AVF), grafts or other) 3) *Laboratory tests*: calcium (mg/dL), phosphorus (mg/dL), serum total cholesterol (mg/dl), and serum albumin (g/dl) 4) *Comorbidity*: ICED score (computed without the diabetes related questions). Since these data were collected in the setting of a randomized trial, we included treatment assignment as covariates (dialysis dose and membrane flux).

### Statistical analysis

In this secondary analysis of the HEMO study data, baseline demographic, socioeconomic and laboratory variables were summarized as mean(standard deviation) for normally distributed continuous variables and as percentages for categorical variables. Skewed continuous variables were summarized using the median ± inter-quartile range. Differences in summary statistics were tested, 1) for the normally distributed variables using a two sided t-test 2) for skewed continuous variables using a rank sum test, and 3) for categorical variables using a chi-square test. To understand the relationship between outcome and the predictors of interest, both the Cox-TVC and Gray’s models were fit to the data. All-cause and cause-specific mortality analyses for cardiovascular, cardiac, and infectious diseases included the following variables in addition to the diabetes variables: age, race, BMI, years of dialysis, Kt/V, flux, dialysis access, ICED, systolic and diastolic blood pressure, smoking, calcium, phosphorus, and serum albumin.

*Cox’s time dependent covariatemodel.* The Cox proportional hazards model depends on the assumption of a constant hazard over time. That means the hazard in the diabetic group is a constant proportion of the hazard in the non-diabetic group. If a time varying covariate is used in a Cox model then proportional hazard assumption violates. While this assumption holds in many different applications, the model can provide misleading results when the hazard function changes over time. To accommodate this possibility, the standard Cox proportional hazards regression model can be extended to fit a model with time varying covariates [7,9]. In Cox-TVC model the values of the covariate may vary over time. For example, diabetic status of an individual may vary over time whereas race is constant over time. We fit single predictor Cox-PH models for each of the variables considered for analysis and tested the proportional hazards assumption. For diabetes, a variable that does not satisfy the proportional hazards assumption, we created a time-varying covariate that included diabetes status by time. We assessed the influence of including other covariate by time interactions in the model and found that there was very little influence in the interpretation of the risk associated with diabetes. Therefore, the more parsimonious model using the diabetes status by time covariate was examined. The final multivariable Cox-TVC model was fit using a significance level of 0.15 as a cutoff for inclusion in the model, with the exception of the variables for study interventions and diastolic blood pressure.

*Gray’s time varying coefficientmodel.* The use of time varying covariate in the Cox model involves the choice of complex functional form of the covariate and requires deep biologic insight. It also may lead to potential bias, and does not lead to prediction for the individual survival experience as does the usual Cox model with fixed covariate values [13]. The statistical formulation of Gray’s model is very similar to the Cox proportional hazards model with the hazard function being a more general version of the function used to fit Cox’s model. For Gray’s model, the regression coefficients differ across set time intervals allowing for the inclusion of non-proportional hazards and providing a better description of the change in risk over time. During each time interval, the rise is assumed to be constant; i.e., the proportionality of hazards is assumed to hold within but not between successive intervals. Assuming that the recorded survival times divided into c + 1 time intervals $\left(0,t_{1}),{(t}_{1},t_{2}),\cdots ,{(t}_{c-1},t_{c}\right),\;\left(t_{c}\right),\;$ where the last interval includes the largest observed time. Then the hazard function for the Gray’s model is given by $\text{h(t,}\;{\text{x) = h}}_{0}\left(t\right)\;\text{exp(x}\;{\text{β}}_{k})$, k=1,⋯,c+1, for t lies between $t_{k-1}$ and $t_{k}$. Based on Gray’s model, there will be $\left(\text{c+}1\right)\;\text{βs}$ associated with each covariate. Gray’s model relies on a flexible spline model to include these changes over time, requiring the specification of the number of time intervals needed to fit the model. The model output then includes a regression coefficient for each of the time intervals, a test of the overall statistical significance of the regression coefficients, and a test of proportionality through a test of the equality of the coefficients over time. The models presented here have been fit using 10 time intervals, so that each interval contains approximately the same number of mortality events [8]. We examined both single predictor Gray’s models as well as models that included multiple predictors. As with the Cox regression models, we used a significance level of 0.15 as inclusion criteria for any variables considered for modeling. The statistical formulation and technical details for Gray’s model can be found in [8,9]. We used Stata 10 and R software for fitting the Cox-TVC model and Gray’s model respectively.

## Results

Baseline characteristics of the HEMO study participants are shown in Table 1. In the HEMO Study, 823 (44.6%) participants were classified as diabetic. Diabetic patients were more likely to be older, female, and have a higher BMI, and were more likely to have tunneled dialysis catheters compared to non-diabetic participants. Diabetics had a higher burden of comorbid disease (higher ICED scores), higher systolic and lower diastolic blood pressure. On the other hand, diabetic patients were less likely to be current smokers and had lower phosphorus. Albumin was significantly lower in the diabetic group compared to non-diabetics. We examined the stability of diabetes classifications over time. In year 1, 17 of the 761 classified as non-diabetic alive at year 1 were reclassified as diabetic. There were no diabetics reclassified as non-diabetics. Hence, 17 of 1316 alive at year 1 were reclassified overall. The diabetes classification remained stable during the rest of the HEMO study period when we examined changes from baseline to years 2–6.

**Table 1:** **Baseline Characteristics of 1846 HEMO Dialysis Patients, USA, 1995-2000**

**Factors**	**All Patients (N =** **1846)**	**Diabetic (N = 823)**	**Non-Diabetic (N = 1023)**	**P-value**^a^
Age	57.62(14.04)	61.19(11.38)	54.75(15.27)	<0.01
Female sex (%)	56.23	63.67	50.24	<0.01
Black race (%)	62.62	64.76	60.90	0.09
BMI	25.46(5.28)	26.86(5.35)	24.35(4.95)	<0.01
Years of dialysis‡	2.16[0.94, 4.68]	1.67[0.81, 3.26]	2.83[1.12, 6.46]	<0.01
Residual urine output (%)^†^	12.02	12.94	11.29	0.29
High Kt/V (%)	49.84	49.70	49.95	0.91
High flux (%)	49.89	50.06	49.76	0.90
Access				
Permanent Catheter (%)	5.80	6.80	4.99	
AVF/AVG/Other (%)	94.20	93.20	95.01	
Co-morbidity ICED score (%)				
0-1	35.59	22.84	45.85	
2	31.26	33.41	29.52	<0.01
3	33.15	43.74	24.63	
Blood pressure				
Systolic	151.02(25.64)	155.92(25.87)	147.08(24.78)	<0.01
Diastolic	81.28(15.24)	79.93(15.01)	82.36(15.35)	<0.01
Smoking (%)				
Never	50.24	54.20	47.06	
Past	32.39	34.10	31.02	
Current	17.36	11.69	21.92	
Calcium (mg/dL)	9.34(0.99)	9.27(0.93)	9.40(1.04)	<0.01
Phosphorus (mg/dL)	5.85(1.83)	5.71(1.76)	5.96(1.89)	0.01
Serum Total Cholesterol(mg/dl)	171.39(40.01)	170.20( 40.48)	172.35(39.62)	0.26
Serum albumin(g/dl)	3.63(0.36)	3.55(0.33)	3.68(0.37)	<0.01

Abbreviations: *BMI*, body mass index; *AVF*, arteriovenous fistula; *AVG*, Arteriovenous grafts; *ICED*, Index of Coexistent Disease.

^a^ All *P* values are two-sided.

^†^If the patient produces ≥200 ml/day of urine, is urea clearance measured from interdialytic urine collection >1.5 ml/min (per 35 L of total urea volume). (0 = no, either produces <200 ml/day or urea clearance ≤ 1.5 ml/min, 1 = yes, 9 = unknown, to be determined during Baseline).

‡ Continuous and skewed variables are summarized in the form of Median [1st Quartile, 3^rd^ Quartile].

The crude hazard estimates obtained from the Cox-PH model and Gray’s model are shown in Table 2. First, unadjusted analyses of all covariates were performed and the proportionality assumption was tested using the Schoenfeld residuals. The residual tests indicate that most of the covariates satisfy the proportional hazards assumption with the exception of diabetes (p = 0.05), duration of dialysis (p = 0.05), systolic blood pressure (p <0.01), smoking (p = 0.03), and albumin (p <0.01). Note, residual urine output violates the PH assumption (p = 0.05) but it was not considered in the multivariable adjusted models since its significance levels are higher than the cutoff point after using residual urine output and time as an interaction term. While the Cox hazard estimate is presented with a p-value derived from the Wald score, the output from Gray’s model provides the range of the hazard ratios across time and the statistical test results from the global test [8] of a nonzero hazard rate. Using the Cox-PH model, the presence of diabetes was associated with a higher risk of death with the HR equal to 1.64; while the HR ranged from 1.46 to 2.21 in Gray’s model. The interpretation of the other hazards associated with the covariates was similar across the two methods with the exception of smoking and systolic blood pressure which were not significant in the Cox-PH model, but had a significant effect when using Gray’s model. Years of dialysis was non-significant in the Cox-PH model but approached significance in Gray’s model (p = 0.08). The HR estimates from the Gray’s models for all time intervals can be found in the supplementary table, “Additional file 1: Table S2”.

**Table 2:** **Baseline Risk Factors and Associated Mortality Using Unadjusted Cox Proportional Hazard (Cox-PH) and Gray’s Survival Models**

**Variables**	**Cox-PH Model**		**PH test**	**Grays Model**	
	**H.R.**^**a**^**Estimate [LL, UL]**	**P-value**	**P-value**	**H.R. Range**^**b**^**(min – max)**	**P-value**
Diabetes	1.64 [1.43, 1.87]	<0.01	0.05	1.46-2.21	<0.01
Age^£^	1.46 [1.38, 1.55]	<0.01	0.98	1.44-1.50	<0.01
Female sex	1.01 [0.88, 1.16]	0.88	0.13	0.91-1.19	0.52
Black race	0.73 [0.64, 0.84]	<0.01	0.09	0.68-0.93	<0.01
BMI	0.98 [0.97, 0.99]	<0.01	0.28	0.96-0.99	<0.01
Yrs of Dialysis	0.99 [0.97, 1.00]	0.18	0.05	0.99-1.01	0.08
Residual urine output	0.97 [0.77, 1.22]	0.80	0.05	0.73-1.22	0.23
≥200 ml/day urine					
High Kt/V	0.95 [0.83, 1.08]	0.41	0.72	0.91-0.96	0.88
High Flux	0.95 [0.83, 1.08]	0.41	0.15	0.87-1.11	0.33
Access(Cather vs. All other)	1.91 [1.47, 2.47]	<0.01	0.79	1.75-2.02	<0.01
Comorbidity ICED Score	1.57 [1.44, 1.70]	<0.01	0.79	1.51-1.67	<0.01
Blood Pressure					
Systolic*	0.97 [0.91, 1.04]	0.37	<0.01	0.99-1.00	0.01
Diastolic*	0.80 [0.75, 0.86]	<0.01	0.38	0.80-0.87	<0.01
Smoking(Smoked vs. Never)	1.08 [0.99, 1.18]	0.09	0.03	0.95-1.18	0.04
Calcium (mg/dL)	0.93 [0.86, 0.99]	0.03	0.08	0.85-1.01	<0.01
Phosphorus (mg/dL)*	0.98 [0.91, 1.05]	0.55	0.49	0.95-1.01	0.87
Total Cholesterol (mg/dL)*	0.99 [0.92, 1.06]	0.78	0.95	0.97-1.02	0.95
Albumin (mg/dL)	0.57 [0.52, 0.63]	<0.01	<0.01	0.44-0.66	<0.01

Abbreviations: *BMI*, body mass index; *ICED*, Index of Coexistent Disease; *PH*, proportional hazard, *H.R.*, hazard ratio.

^a^ Hazard Ratio (HR) estimate for each risk factor is presented in the second column which is obtained by fitting the Cox’s time varying single covariate model.

^b^ A range of HR estimates are obtained for each covariate by fitting Gray’s model. £ Age has been rescaled by dividing 10 for better interpretation of the HR.

*Blood pressure, phosphorus, and total cholesterol have been rescaled by dividing standard deviation for better interpretation of the HR.

The adjusted hazards for all-cause mortality from both the Cox and Gray’s survival models are presented in Table 3. After fitting the multivariable standard Cox-PH model for all causes mortality survival data, the Schoenfeld residuals test indicated that the model violated the proportional hazard assumption (global test, p <0.01; diabetes, p = 0.07). In the Cox model with time-varying covariates the hazard ratio (HR) for diabetes is 1.57, p = 0.11, and the interaction between diabetes and time has a HR of 1.13 with p = 0.02. The combined test for diabetes and diabetes × time using the likelihood ratio test was significant with a chi-square = 7.04 and p = 0.03. At the 1^st^ and 6^th^ year the hazard ratios are 1.77 and 3.22 respectively. When systolic blood pressure was included in the model using an interaction term with time, there was no substantial change in the hazard for diabetes. Using Gray’s model the HR for diabetes ranges from 1.41 to 2.21 with p <0.01. In the multi-predictor models of all-cause mortality, the estimates for other covariates were similar for most of the covariates. The corresponding p-values for the variables BMI, years of dialysis, smoking, and phosphorus are very similar for these two models (Table 3). There were some slight differences in the interpretation of the coefficients between the two models. For example, systolic blood pressure was associated with a HR of 0.98 using the Cox model and the estimate of the HR with Gray’s model ranged from 0.88-1.07. For serum calcium which was not statistically significant (at 5% level) using Cox’s model with time-varying covariates, the results obtained from Gray’s model indicated that the relationship was significant with the HR ranging from 0.87-1.00. The HR estimates from the Gray’s model for all time intervals can be found in the supplementary table, “Additional file 2: Table S3”.

**Table 3:** **Multi-Predictor Cox and Gray’s Survival Models for HEMO Dialysis Patients’ Population**

**Variables**	**Cox-TVC Model**		**Grays Model**	
	**H.R.**	**P-value**	**H.R. Range (min-max)**	**P-value**
Diabetes	1.57 [0.91, 2.73]	0.11	1.41-2.21	<0.01
Diabetes × time	1.13 [1.02, 1.25]	0.02	--	--
Age	1.44 [1.34, 1.55]	0.00	1.41-1.48	<0.01
Female Sex	1.08 [0.87, 1.34]	0.51	0.99-1.23	0.86
Diabetes × Sex	0.75 [0.55, 1.01]	0.06	0.59-0.85	0.05
Black Race	0.72 [0.62, 0.84]	<0.01	0.65-0.83	<0.01
BMI	0.97 [0.95, 0.98]	<0.01	0.95-0.99	<0.01
Yrs of Dialysis	1.03 [1.01, 1.04]	0.01	1.01-1.04	0.01
High Kt/V	0.97 [0.83, 1.12]	0.64	0.89-1.04	0.84
High Flux	0.94 [0.81, 1.09]	0.43	0.83-1.23	0.11
Access(Catheter vs. All other)	1.67 [1.24, 2.24]	<0.01	1.41-2.00	<0.01
Comorbidity ICED Score	1.39 [1.26, 1.53]	<0.01	1.23-1.55	<0.01
Blood Pressure				
Systolic	0.98 [0.89, 1.09]	0.74	0.88-1.07	0.07
Diastolic	0.92 [0.83, 1.02]	0.12	0.89-0.95	0.30
Smoking (Smoked vs. Never)	1.15 [1.03, 1.27]	0.01	1.03-1.24	0.01
Calcium(mg/dL)	0.93 [0.87, 1.00]	0.07	0.87-1.00	0.04
Phosphorus(mg/dL)	1.12 [1.03, 1.21]	0.01	1.09-1.15	0.02
Albumin(mg/dL)	0.70 [0.62, 0.78]	<0.01	0.61-0.77	<0.01

Abbreviations: *BMI,* body mass index; *AVF*, arteriovenous fistula; *AVG*, Arteriovenous grafts; *ICED*, Index of Coexistent Disease, *H.R.*, hazard ratio.

**Figure 1:**
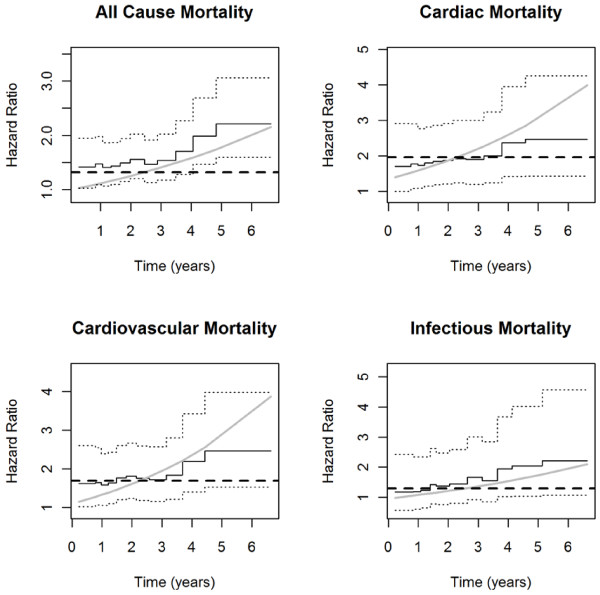
**All-cause and Cause-specific Adjusted Hazard Ratio for Diabetes Over Time in HEMO Study Patients**^**§§**^**.**^**§§**^ (**a**) **All-cause mortality** HR estimate using the Cox’s proportional hazards (Cox-PH) is 2.11 (p = 0.003), HR for DM using Cox-TVC model is 1.57 (p = 0.11) for main effects and 1.13 (p = 0.02) for the interaction term, Gray’s model estimates are 1.41 to 2.21 (p <0.001). (**b**) **Cardiac mortality** HR estimate using the Cox-PH is 1.81 (p = 0.16), HR for DM using Cox-TVC model is 1.22 (p = 0.68) for main effects and 1.18 (p = 0.07) for the interaction term, Gray’s model estimates are 1.70 to 2.46 (p = 0.003). (**c**) **Cardiovascular mortality** HR estimate using the Cox-PH is 2.00 (p = 0.06), HR for DM using Cox-TVC model is 1.28 (p = 0.55) for main effects and 1.21 (p = 0.02) for the interaction term, Gray’s model estimates are 1.58 to 2.46 (p = 0.001). (**d**) **Infectious mortality** HR estimate using the Cox-PH is 2.01 (p = 0.22), HR for DM using Cox-TVC model is 1.48 (p = 0.53) for main effects and 1.12 (p = 0.27) for the interaction term, Gray’s model estimates are 1.17 to 2.21 (p = 0.21).

Figure 1 demonstrates the influence of diabetes status for all-cause and cause- specific mortality for diabetes as estimated from each of the three models; the Cox-PH model(dash line), the Cox model with time-varying covariates(light solid line) and Gray’s model(solid line including 95% confidence interval). While diabetes violates the PH assumption for all-cause mortality, it does not violate the PH assumption for the three cause-specific mortalities (Schoenfeld residuals test, p >0.05). For better comparison, we have plotted the hazard ratios from the Cox-PH model which is basically a straight line intercepting with y-axis at different levels depending on the types of mortality considered. For the Cox model with time-varying covariates, the plot includes the hazard ratio associated with both the diabetes and diabetes × time interaction terms. For Gray’s model the confidence interval associated with each hazard ratio is also provided. For all-cause mortality (panel a), the Cox-TVC model provides an estimate of risk below that of the Gray’s method. Both the Cox-TVC and Gray’s method demonstrated a higher long-term risk associated with diabetes than the adjusted Cox proportional hazards model. For cardiac mortality (panel b), the Cox proportional hazard ratio estimate for diabetes was 1.81, 95% CI [0.79, 4.15] (Schoenfeld residuals test, p = 0.30). Again, there was not a significant interaction with time (p = 0.07) for the Cox-TVC, and Gray’s method provided a very similar estimate of risk for the first four years in the study and the risk increased in years 5 and 6. For cardiovascular mortality(panel c), the Cox-TVC model provides an estimate of risk below that of Gray’s method for the first two years, then Gray’s method and the Cox-TVC are approximately similar through five years. Both the Cox-TVC and the Gray’s method demonstrated a higher long-term risk associated with diabetes than the adjusted Cox proportional hazards model. For infectious mortality (panel d), the hazards estimated for diabetes from the three methods appear similar for the first 4 years then estimates differ depending on the methods used.

## Discussion

This work demonstrated that diabetes was associated with an increasing risk of cardiac, cardiovascular and all-cause mortality over time in the HEMO study. There was no increase in risk from infectious mortality associated with diabetes. The use of the HEMO study to assess the risk of death in the hemodialysis population takes advantage of a randomized, controlled dialysis trial with stratification using diabetes and provides the opportunity to account for the level of comorbidity using the ICED. This ability to adjust for differences was important since there were significant differences in the distribution of characteristics by diabetes in HEMO. These findings demonstrate that the risk of death associated with diabetes in ESRD increases over time and suggest that an increasing risk of death among diabetes may be underappreciated when using conventional survival models.

These findings extend earlier work demonstrating an increased risk of death from diabetes among both hemodialysis patients and within the general population [14,15]. While ESRD confers an increased risk of mortality [16], previous research has shown that the risk of death is even higher among ESRD patients with diabetes when compared to non-diabetic ESRD patients, indicating that diabetes confers an independent mortality risk [17-19]. Villar et al. analyzed the data from the ANZ Dialysis and Transplant Registry and assessed the risk factors for death using Cox regression demonstrating that the adjusted hazard ratio of death was 1.64 in type 1 diabetics and 1.13 in type 2 diabetics compared to non-diabetic patients with ESRD [3]. Van DijK et al. analyzed the data from 10 registries in Europe and found the diabetes was associated with an increased risk of death in ESRD population [2]. A comparison of outcomes between diabetic and non-diabetic CAPD patients was performed by Prasad et al.; this study showed that diabetes was associated with an odds ratio of 1.95 (CI 1.23-3.07) for overall mortality [20]. Survival of patients on maintenance hemodialysis over a twenty year period was analyzed by Sikole et al. who showed that hemodialysis patients with diabetes had a lower survival rate compared to patients with glomerulonephritis and adult polycystic kidney disease [21]. Since the Membrane Permeability Outcome (MPO) study demonstrated that diabetic patients in the high flux arm had better survival probability than the diabetic patients in the low flux arm [22], it is important that these findings from the HEMO study accounted for assignment to the high flux arm. The increased risk of death associated with diabetes in the HEMO study was due to cardiac and cardiovascular mortality rather than infectious mortality.

These findings from the HEMO study demonstrate an increasing risk of mortality over time associated with diabetes in prevalent hemodialysis patients which may relate to the accumulation of end-organ damage or mediators of inflammation and oxidative stress. The accumulation of advanced glycosylated products over the course of many years can theoretically be responsible for an increasing risk of all-cause and cardiovascular mortality due to diabetes over time. The pathophysiological basis of structural and functional changes due to diabetes which may lead to endothelial dysfunction and increased cardiovascular risk is thought to involve oxidative stress and production of advanced glycosylated products [23-25]. Previous research studying the effect of diabetes on mortality in ESRD patients have assumed that the risk of mortality (or hazard ratio) due to diabetes remains constant over time and have utilized the Cox proportional hazards model to estimate the effects of diabetes. However, diabetes may violate the proportionality assumption required by the Cox model, leading to an inaccurate estimate of risk. In this situation alternative approaches, such as Cox models that include time interactions (TVC) or Gray’s [8] extension of the Cox model may be used to better estimate the risk associated with diabetes. Gray’s modeling technique has been applied to many other areas of biomedical research with the reporting of survival probabilities [9]. In sum, this work suggests that using the standard survival model underestimates the burden of diabetes among hemodialysis patients in the HEMO study.

These findings should be interpreted in light of several limitations. Gray’s model requires estimation of a large number of coefficients (default is 10) for a variable in the model and hence it requires larger sample sizes [9]. This limitation was overcome by our selection of the HEMO Study, which is a large trial of hemodialysis patients. In our comparison of two models: Cox-TVC and Gray’s model, we do not have a ‘gold standard’ model for comparison. Also, the HEMO study studied prevalent hemodialysis patients rather than incident patients which could create a survivorship bias. However, the mortality rates in HEMO were similar to those seen in the USRDS cohort [6]. Also, the use of prevalent patients addresses an important subgroup of hemodialysis patients since while the adjusted mortality rates have declined over the past two-decades for patients exposed to less than 5-years of dialysis, those patients with greater than 5-years of dialysis exposure have not experienced these gains in survival [26]. Furthermore, the available source of data did not allow us to make an accurate distinction between Type 1 and Type 2 diabetes, so that potential differences between diabetes subtypes are not accounted for in this analysis.

## Conclusions

Our findings of increasing risk of all-cause mortality and cardiovascular and cardiac mortality demonstrate that the burden of diabetes increases over time among hemodialysis patients. This finding is important for understanding the burden of diabetes among patients with ESRD and may contribute to our interpretation of the pathophysiology of diabetes. This work also builds on reviews of time-to-event analysis in ESRD by applying flexible survival approaches to better understand outcomes among the ESRD population [27]. These approaches provide more precise estimates for the outcomes of diabetics undergoing hemodialysis. Using this information may permit the patient to weigh the decision to start dialysis or to consider evaluation for kidney transplantation. Further studies could combine novel methods for survival analysis with longitudinal assessment of biomarkers such as C- reactive protein or markers of oxidative stress to better understand the strong association of diabetes with an increasing risk of death in prevalent hemodialysis patients.

## Competing interests

This work was sponsored by the Baxter Healthcare Extramural Grant Program. The sponsor of the study had no role in the study design, data collection, data analysis, interpretation of the study results, or writing of the manuscript. The corresponding author had full access to all the data and coordinated the decision to submit for publication.

## Authors' contributions

In this study Author AS compared and fitted survival models, wrote abstract, introduction, statistical analysis, results, and discussion. Author CA helped in determining appropriate survival models for the comparison and created a graph for the hazard ratio and participated in writing of the manuscript. Author LW helped intellectually in developing the study, in revision of the text, and with the statistical analysis. Author NY formed the original study questions, performed the literature review and contributed to the text. Author LF helped intellectually, and revised and critiqued various points for the improvement. Author JAK helped intellectually, and helped in revision of the text. Author MU designed the study and directed its implementation including writing and revision of the text. All authors read and approved the final manuscript.

## Pre-publication history

The pre-publication history for this paper can be accessed here:

http://www.biomedcentral.com/1471-2369/13/130/prepub

## Supplementary Material

Additional file 1 **Table S2.** Detail HR estimates from the Gray’s models for all time intervals. Click here for file

Additional file 2 **Table S3.** Detail HR estimates from the Gray’s model for all time intervals. Click here for file
