# Supplementary material for: All-cause and cause-specific mortality associated with diabetes in prevalent hemodialysis patients
Source: BMC Nephrol. 2012 Oct 1;13:130. doi: 10.1186/1471-2369-13-130 (PMC3519533; doi:10.1186/1471-2369-13-130)
Supplement: Additional file 1 — Table S2. Detail HR estimates from the Gray’s models for all time intervals. [file 1471-2369-13-130-S1.doc]

**Supplementary table**

Table S2: Detail HR estimates from the Gray's models for all time intervals

| **Variables** | **Time(Yrs)** | **Coeff** | **Std.Err** | **HR** | **^$^LL** | **^$^UL** | **p-value** |
| --- | --- | --- | --- | --- | --- | --- | --- |
| High Kt/V | 0.003 | -0.092 | 0.108 | 0.912 | 0.738 | 1.126 | 0.39 |
| High Kt/V | 0.444 | -0.069 | 0.098 | 0.934 | 0.771 | 1.131 | 0.48 |
| High Kt/V | 0.794 | -0.055 | 0.089 | 0.947 | 0.795 | 1.128 | 0.54 |
| High Kt/V | 1.084 | -0.047 | 0.084 | 0.954 | 0.809 | 1.125 | 0.58 |
| High Kt/V | 1.399 | -0.081 | 0.082 | 0.922 | 0.786 | 1.082 | 0.32 |
| High Kt/V | 1.736 | -0.046 | 0.081 | 0.955 | 0.815 | 1.119 | 0.57 |
| High Kt/V | 2.160 | -0.073 | 0.082 | 0.930 | 0.792 | 1.091 | 0.37 |
| High Kt/V | 2.677 | -0.046 | 0.084 | 0.955 | 0.81 | 1.127 | 0.59 |
| High Kt/V | 3.294 | -0.036 | 0.09 | 0.964 | 0.809 | 1.15 | 0.69 |
| High Kt/V | 3.962 | -0.038 | 0.098 | 0.963 | 0.794 | 1.167 | 0.70 |
| High Kt/V | 4.750 | -0.037 | 0.108 | 0.963 | 0.78 | 1.19 | 0.73 |
|  |  |  |  |  |  |  |  |
| High Flux | 0.003 | -0.14 | 0.108 | 0.869 | 0.704 | 1.074 | 0.19 |
| High Flux | 0.444 | -0.115 | 0.098 | 0.891 | 0.736 | 1.08 | 0.24 |
| High Flux | 0.793 | -0.108 | 0.09 | 0.898 | 0.753 | 1.07 | 0.23 |
| High Flux | 1.084 | -0.081 | 0.084 | 0.922 | 0.781 | 1.087 | 0.33 |
| High Flux | 1.399 | -0.079 | 0.082 | 0.924 | 0.788 | 1.085 | 0.34 |
| High Flux | 1.735 | -0.108 | 0.081 | 0.898 | 0.766 | 1.052 | 0.18 |
| High Flux | 2.160 | -0.101 | 0.082 | 0.904 | 0.77 | 1.061 | 0.22 |
| High Flux | 2.677 | -0.069 | 0.084 | 0.934 | 0.791 | 1.101 | 0.42 |
| High Flux | 3.293 | 0 | 0.09 | 1.000 | 0.839 | 1.192 | 1.00 |
| High Flux | 3.961 | 0.079 | 0.098 | 1.082 | 0.893 | 1.311 | 0.42 |
| High Flux | 4.750 | 0.105 | 0.108 | 1.111 | 0.899 | 1.372 | 0.33 |
|  |  |  |  |  |  |  |  |
| Age | 0.003 | 0.401 | 0.046 | 1.494 | 1.364 | 1.636 | 0.00 |
| Age | 0.444 | 0.387 | 0.042 | 1.473 | 1.356 | 1.6 | 0.00 |
| Age | 0.794 | 0.388 | 0.038 | 1.474 | 1.367 | 1.59 | 0.00 |
| Age | 1.084 | 0.362 | 0.036 | 1.436 | 1.338 | 1.542 | 0.00 |
| Age | 1.399 | 0.378 | 0.035 | 1.460 | 1.363 | 1.563 | 0.00 |
| Age | 1.736 | 0.367 | 0.034 | 1.444 | 1.349 | 1.544 | 0.00 |
| Age | 2.160 | 0.364 | 0.035 | 1.439 | 1.344 | 1.541 | 0.00 |
| Age | 2.677 | 0.384 | 0.036 | 1.468 | 1.368 | 1.575 | 0.00 |
| Age | 3.294 | 0.379 | 0.038 | 1.460 | 1.355 | 1.573 | 0.00 |
| Age | 3.962 | 0.382 | 0.042 | 1.465 | 1.35 | 1.59 | 0.00 |
| Age | 4.750 | 0.404 | 0.046 | 1.497 | 1.368 | 1.639 | 0.00 |
|  |  |  |  |  |  |  |  |
| Black race | 0.003 | -0.374 | 0.11 | 0.688 | 0.555 | 0.853 | 0.00 |
| Black race | 0.444 | -0.361 | 0.099 | 0.697 | 0.574 | 0.847 | 0.00 |
| Black race | 0.794 | -0.393 | 0.091 | 0.675 | 0.565 | 0.806 | 0.00 |
| Black race | 1.084 | -0.34 | 0.086 | 0.712 | 0.602 | 0.842 | 0.00 |
| Black race | 1.399 | -0.332 | 0.083 | 0.718 | 0.61 | 0.845 | 0.00 |
| Black race | 1.736 | -0.374 | 0.083 | 0.688 | 0.585 | 0.809 | 0.00 |
| Black race | 2.160 | -0.361 | 0.084 | 0.697 | 0.591 | 0.822 | 0.00 |
| Black race | 2.677 | -0.346 | 0.088 | 0.707 | 0.596 | 0.84 | 0.00 |
| Black race | 3.294 | -0.256 | 0.094 | 0.774 | 0.644 | 0.93 | 0.01 |
| Black race | 3.962 | -0.14 | 0.103 | 0.870 | 0.711 | 1.064 | 0.18 |
| Black race | 4.750 | -0.07 | 0.113 | 0.932 | 0.747 | 1.164 | 0.54 |
|  |  |  |  |  |  |  |  |
| BMI | 0.003 | -0.041 | 0.011 | 0.960 | 0.939 | 0.981 | 0.00 |
| BMI | 0.444 | -0.028 | 0.01 | 0.972 | 0.953 | 0.992 | 0.01 |
| BMI | 0.794 | -0.025 | 0.009 | 0.976 | 0.958 | 0.994 | 0.01 |
| BMI | 1.084 | -0.019 | 0.009 | 0.981 | 0.965 | 0.998 | 0.03 |
| BMI | 1.399 | -0.011 | 0.008 | 0.989 | 0.972 | 1.005 | 0.17 |
| BMI | 1.736 | -0.013 | 0.008 | 0.987 | 0.971 | 1.003 | 0.12 |
| BMI | 2.160 | -0.02 | 0.008 | 0.981 | 0.965 | 0.997 | 0.02 |
| BMI | 2.677 | -0.022 | 0.009 | 0.979 | 0.962 | 0.995 | 0.01 |
| BMI | 3.294 | -0.022 | 0.009 | 0.979 | 0.961 | 0.996 | 0.02 |
| BMI | 3.962 | -0.02 | 0.01 | 0.980 | 0.961 | 0.999 | 0.04 |
| BMI | 4.750 | -0.015 | 0.011 | 0.985 | 0.964 | 1.007 | 0.18 |
|  |  |  |  |  |  |  |  |
| Yrs of Dialysis | 0.003 | 0.008 | 0.012 | 1.008 | 0.984 | 1.032 | 0.53 |
| Yrs of Dialysis | 0.444 | 0.002 | 0.011 | 1.002 | 0.981 | 1.024 | 0.85 |
| Yrs of Dialysis | 0.794 | -0.003 | 0.01 | 0.997 | 0.977 | 1.017 | 0.74 |
| Yrs of Dialysis | 1.084 | -0.002 | 0.01 | 0.998 | 0.979 | 1.017 | 0.80 |
| Yrs of Dialysis | 1.399 | -0.012 | 0.009 | 0.988 | 0.97 | 1.007 | 0.22 |
| Yrs of Dialysis | 1.736 | -0.013 | 0.009 | 0.987 | 0.969 | 1.005 | 0.16 |
| Yrs of Dialysis | 2.160 | -0.01 | 0.009 | 0.990 | 0.972 | 1.009 | 0.30 |
| Yrs of Dialysis | 2.677 | -0.012 | 0.01 | 0.988 | 0.969 | 1.007 | 0.21 |
| Yrs of Dialysis | 3.294 | -0.017 | 0.01 | 0.984 | 0.964 | 1.004 | 0.11 |
| Yrs of Dialysis | 3.962 | -0.022 | 0.011 | 0.978 | 0.956 | 1 | 0.05 |
| Yrs of Dialysis | 4.750 | -0.033 | 0.012 | 0.968 | 0.945 | 0.992 | 0.01 |
|  |  |  |  |  |  |  |  |
| Access (Cather vs. All other) | 0.003 | 0.703 | 0.196 | 2.020 | 1.376 | 2.966 | 0.00 |
| Access (Cather vs. All other) | 0.444 | 0.69 | 0.175 | 1.994 | 1.415 | 2.81 | 0.00 |
| Access (Cather vs. All other) | 0.794 | 0.639 | 0.161 | 1.894 | 1.38 | 2.599 | 0.00 |
| Access (Cather vs. All other) | 1.084 | 0.562 | 0.156 | 1.754 | 1.292 | 2.379 | 0.00 |
| Access (Cather vs. All other) | 1.399 | 0.603 | 0.156 | 1.828 | 1.347 | 2.481 | 0.00 |
| Access (Cather vs. All other) | 1.736 | 0.629 | 0.16 | 1.876 | 1.371 | 2.568 | 0.00 |
| Access (Cather vs. All other) | 2.160 | 0.68 | 0.168 | 1.974 | 1.419 | 2.746 | 0.00 |
| Access (Cather vs. All other) | 2.677 | 0.67 | 0.182 | 1.953 | 1.367 | 2.791 | 0.00 |
| Access (Cather vs. All other) | 3.294 | 0.697 | 0.2 | 2.009 | 1.356 | 2.975 | 0.00 |
| Access (Cather vs. All other) | 3.962 | 0.578 | 0.221 | 1.783 | 1.157 | 2.747 | 0.01 |
| Access (Cather vs. All other) | 4.750 | 0.59 | 0.235 | 1.803 | 1.137 | 2.859 | 0.01 |
|  |  |  |  |  |  |  |  |
| Comorbidity ICED Score | 0.003 | 0.49 | 0.067 | 1.632 | 1.431 | 1.861 | 0.00 |
| Comorbidity ICED Score | 0.444 | 0.45 | 0.061 | 1.568 | 1.391 | 1.767 | 0.00 |
| Comorbidity ICED Score | 0.794 | 0.427 | 0.056 | 1.533 | 1.374 | 1.709 | 0.00 |
| Comorbidity ICED Score | 1.084 | 0.415 | 0.052 | 1.514 | 1.367 | 1.678 | 0.00 |
| Comorbidity ICED Score | 1.399 | 0.414 | 0.051 | 1.513 | 1.371 | 1.671 | 0.00 |
| Comorbidity ICED Score | 1.736 | 0.41 | 0.05 | 1.507 | 1.366 | 1.662 | 0.00 |
| Comorbidity ICED Score | 2.160 | 0.434 | 0.05 | 1.544 | 1.399 | 1.704 | 0.00 |
| Comorbidity ICED Score | 2.677 | 0.432 | 0.052 | 1.540 | 1.391 | 1.705 | 0.00 |
| Comorbidity ICED Score | 3.294 | 0.463 | 0.055 | 1.589 | 1.427 | 1.77 | 0.00 |
| Comorbidity ICED Score | 3.962 | 0.489 | 0.06 | 1.631 | 1.449 | 1.835 | 0.00 |
| Comorbidity ICED Score | 4.750 | 0.513 | 0.066 | 1.671 | 1.467 | 1.902 | 0.00 |
| Blood Pressure |  |  |  |  |  |  |  |
| Systolic | 0.003 | -0.005 | 0.002 | 0.995 | 0.991 | 0.999 | 0.03 |
| Systolic | 0.444 | -0.005 | 0.002 | 0.995 | 0.991 | 0.999 | 0.01 |
| Systolic | 0.794 | -0.005 | 0.002 | 0.995 | 0.991 | 0.998 | 0.00 |
| Systolic | 1.084 | -0.005 | 0.002 | 0.995 | 0.992 | 0.999 | 0.01 |
| Systolic | 1.399 | -0.004 | 0.002 | 0.996 | 0.993 | 0.999 | 0.02 |
| Systolic | 1.736 | -0.002 | 0.002 | 0.998 | 0.995 | 1.001 | 0.27 |
| Systolic | 2.160 | 0 | 0.002 | 1.000 | 0.997 | 1.004 | 0.77 |
| Systolic | 2.677 | 0.002 | 0.002 | 1.002 | 0.999 | 1.005 | 0.25 |
| Systolic | 3.294 | 0.002 | 0.002 | 1.002 | 0.999 | 1.006 | 0.19 |
| Systolic | 3.962 | 0.003 | 0.002 | 1.003 | 0.999 | 1.007 | 0.11 |
| Systolic | 4.750 | 0.004 | 0.002 | 1.004 | 1 | 1.008 | 0.06 |
|  |  |  |  |  |  |  |  |
| Diastolic | 0.003 | -0.201 | 0.058 | 0.818 | 0.73 | 0.917 | 0.00 |
| Diastolic | 0.444 | -0.227 | 0.053 | 0.797 | 0.718 | 0.883 | 0.00 |
| Diastolic | 0.794 | -0.267 | 0.048 | 0.766 | 0.697 | 0.841 | 0.00 |
| Diastolic | 1.084 | -0.256 | 0.045 | 0.774 | 0.708 | 0.846 | 0.00 |
| Diastolic | 1.399 | -0.277 | 0.044 | 0.758 | 0.695 | 0.827 | 0.00 |
| Diastolic | 1.736 | -0.256 | 0.044 | 0.774 | 0.71 | 0.843 | 0.00 |
| Diastolic | 2.160 | -0.226 | 0.044 | 0.798 | 0.731 | 0.87 | 0.00 |
| Diastolic | 2.677 | -0.215 | 0.046 | 0.807 | 0.737 | 0.883 | 0.00 |
| Diastolic | 3.294 | -0.187 | 0.049 | 0.830 | 0.754 | 0.913 | 0.00 |
| Diastolic | 3.962 | -0.153 | 0.054 | 0.858 | 0.772 | 0.954 | 0.01 |
| Diastolic | 4.750 | -0.138 | 0.059 | 0.871 | 0.776 | 0.978 | 0.02 |
|  |  |  |  |  |  |  |  |
| Smoking (Smoked vs. Never) | 0.003 | 0.168 | 0.07 | 1.183 | 1.032 | 1.357 | 0.02 |
| Smoking (Smoked vs. Never) | 0.444 | 0.151 | 0.063 | 1.163 | 1.027 | 1.317 | 0.02 |
| Smoking (Smoked vs. Never) | 0.794 | 0.147 | 0.058 | 1.158 | 1.034 | 1.298 | 0.01 |
| Smoking (Smoked vs. Never) | 1.084 | 0.128 | 0.055 | 1.137 | 1.021 | 1.265 | 0.02 |
| Smoking (Smoked vs. Never) | 1.399 | 0.119 | 0.053 | 1.127 | 1.015 | 1.25 | 0.03 |
| Smoking (Smoked vs. Never) | 1.736 | 0.097 | 0.053 | 1.102 | 0.994 | 1.222 | 0.07 |
| Smoking (Smoked vs. Never) | 2.160 | 0.06 | 0.053 | 1.062 | 0.956 | 1.179 | 0.26 |
| Smoking (Smoked vs. Never) | 2.677 | 0.047 | 0.055 | 1.048 | 0.941 | 1.168 | 0.39 |
| Smoking (Smoked vs. Never) | 3.294 | -0.012 | 0.059 | 0.988 | 0.88 | 1.108 | 0.84 |
| Smoking (Smoked vs. Never) | 3.962 | -0.023 | 0.064 | 0.978 | 0.862 | 1.109 | 0.73 |
| Smoking (Smoked vs. Never) | 4.750 | -0.056 | 0.071 | 0.946 | 0.823 | 1.086 | 0.43 |
|  |  |  |  |  |  |  |  |
| Calcium | 0.279 | -0.161 | 0.056 | 0.851 | 0.763 | 0.949 | 0.00 |
| Calcium | 0.832 | -0.153 | 0.051 | 0.858 | 0.777 | 0.947 | 0.00 |
| Calcium | 1.095 | -0.153 | 0.046 | 0.858 | 0.784 | 0.94 | 0.00 |
| Calcium | 1.379 | -0.148 | 0.044 | 0.862 | 0.792 | 0.939 | 0.00 |
| Calcium | 1.664 | -0.095 | 0.042 | 0.909 | 0.837 | 0.987 | 0.02 |
| Calcium | 2.017 | -0.027 | 0.042 | 0.974 | 0.897 | 1.056 | 0.52 |
| Calcium | 2.510 | 0.008 | 0.042 | 1.008 | 0.929 | 1.094 | 0.84 |
| Calcium | 2.962 | 0.004 | 0.043 | 1.004 | 0.923 | 1.091 | 0.93 |
| Calcium | 3.529 | -0.027 | 0.045 | 0.974 | 0.892 | 1.063 | 0.56 |
| Calcium | 4.068 | -0.047 | 0.049 | 0.954 | 0.867 | 1.05 | 0.33 |
| Calcium | 4.829 | -0.074 | 0.054 | 0.928 | 0.835 | 1.032 | 0.17 |
|  |  |  |  |  |  |  |  |
| Phosphorus | 0.279 | 0.006 | 0.058 | 1.006 | 0.898 | 1.128 | 0.91 |
| Phosphorus | 0.832 | 0.003 | 0.053 | 1.003 | 0.904 | 1.112 | 0.96 |
| Phosphorus | 1.095 | 0.004 | 0.048 | 1.004 | 0.914 | 1.104 | 0.93 |
| Phosphorus | 1.379 | 0.003 | 0.046 | 1.003 | 0.917 | 1.097 | 0.95 |
| Phosphorus | 1.664 | -0.025 | 0.044 | 0.976 | 0.895 | 1.064 | 0.58 |
| Phosphorus | 2.017 | -0.051 | 0.044 | 0.950 | 0.872 | 1.036 | 0.25 |
| Phosphorus | 2.510 | -0.056 | 0.045 | 0.946 | 0.866 | 1.033 | 0.21 |
| Phosphorus | 2.962 | -0.044 | 0.047 | 0.957 | 0.874 | 1.049 | 0.35 |
| Phosphorus | 3.529 | -0.033 | 0.05 | 0.968 | 0.878 | 1.066 | 0.51 |
| Phosphorus | 4.082 | -0.026 | 0.054 | 0.974 | 0.876 | 1.084 | 0.64 |
| Phosphorus | 4.829 | -0.031 | 0.06 | 0.969 | 0.862 | 1.09 | 0.60 |
|  |  |  |  |  |  |  |  |
| Albumin (mg/dL) | 0.003 | -0.812 | 0.077 | 0.444 | 0.382 | 0.517 | 0.00 |
| Albumin (mg/dL) | 0.444 | -0.73 | 0.07 | 0.482 | 0.42 | 0.553 | 0.00 |
| Albumin (mg/dL) | 0.794 | -0.654 | 0.064 | 0.520 | 0.459 | 0.59 | 0.00 |
| Albumin (mg/dL) | 1.084 | -0.614 | 0.061 | 0.541 | 0.481 | 0.61 | 0.00 |
| Albumin (mg/dL) | 1.399 | -0.584 | 0.059 | 0.558 | 0.497 | 0.626 | 0.00 |
| Albumin (mg/dL) | 1.736 | -0.52 | 0.059 | 0.594 | 0.53 | 0.667 | 0.00 |
| Albumin (mg/dL) | 2.160 | -0.451 | 0.06 | 0.637 | 0.567 | 0.716 | 0.00 |
| Albumin (mg/dL) | 2.677 | -0.416 | 0.062 | 0.660 | 0.585 | 0.745 | 0.00 |
| Albumin (mg/dL) | 3.294 | -0.445 | 0.066 | 0.641 | 0.564 | 0.729 | 0.00 |
| Albumin (mg/dL) | 3.962 | -0.46 | 0.072 | 0.631 | 0.548 | 0.727 | 0.00 |
| Albumin (mg/dL) | 4.750 | -0.47 | 0.079 | 0.625 | 0.535 | 0.73 | 0.00 |
|  |  |  |  |  |  |  |  |
| Diabetes | 0.003 | 0.452 | 0.108 | 1.571 | 1.271 | 1.942 | 0.00 |
| Diabetes | 0.444 | 0.446 | 0.098 | 1.562 | 1.289 | 1.894 | 0.00 |
| Diabetes | 0.794 | 0.41 | 0.09 | 1.506 | 1.263 | 1.796 | 0.00 |
| Diabetes | 1.084 | 0.375 | 0.084 | 1.455 | 1.233 | 1.717 | 0.00 |
| Diabetes | 1.399 | 0.415 | 0.082 | 1.514 | 1.29 | 1.777 | 0.00 |
| Diabetes | 1.736 | 0.44 | 0.081 | 1.553 | 1.325 | 1.82 | 0.00 |
| Diabetes | 2.160 | 0.425 | 0.082 | 1.530 | 1.303 | 1.796 | 0.00 |
| Diabetes | 2.677 | 0.454 | 0.085 | 1.574 | 1.334 | 1.858 | 0.00 |
| Diabetes | 3.294 | 0.55 | 0.09 | 1.733 | 1.453 | 2.067 | 0.00 |
| Diabetes | 3.962 | 0.686 | 0.098 | 1.986 | 1.637 | 2.408 | 0.00 |
| Diabetes | 4.750 | 0.793 | 0.108 | 2.211 | 1.788 | 2.734 | 0.00 |
|  |  |  |  |  |  |  |  |
| Female Sex | 0.003 | -0.099 | 0.108 | 0.906 | 0.733 | 1.12 | 0.36 |
| Female Sex | 0.444 | -0.052 | 0.098 | 0.949 | 0.783 | 1.151 | 0.60 |
| Female Sex | 0.794 | -0.059 | 0.09 | 0.943 | 0.791 | 1.125 | 0.51 |
| Female Sex | 1.084 | -0.039 | 0.085 | 0.961 | 0.814 | 1.135 | 0.64 |
| Female Sex | 1.399 | -0.017 | 0.082 | 0.983 | 0.837 | 1.154 | 0.83 |
| Female Sex | 1.736 | -0.008 | 0.081 | 0.992 | 0.846 | 1.163 | 0.92 |
| Female Sex | 2.160 | 0.004 | 0.082 | 1.004 | 0.855 | 1.18 | 0.96 |
| Female Sex | 2.677 | 0.017 | 0.085 | 1.017 | 0.861 | 1.201 | 0.85 |
| Female Sex | 3.294 | 0.061 | 0.09 | 1.063 | 0.891 | 1.269 | 0.50 |
| Female Sex | 3.962 | 0.134 | 0.099 | 1.143 | 0.942 | 1.387 | 0.18 |
| Female Sex | 4.750 | 0.176 | 0.109 | 1.192 | 0.963 | 1.475 | 0.11 |
|  |  |  |  |  |  |  |  |
| Total Cholesterol (mg/Dl) | 0.003 | -0.025 | 0.057 | 0.975 | 0.872 | 1.091 | 0.66 |
| Total Cholesterol (mg/Dl) | 0.444 | -0.029 | 0.052 | 0.971 | 0.877 | 1.075 | 0.57 |
| Total Cholesterol (mg/Dl) | 0.794 | -0.032 | 0.047 | 0.968 | 0.882 | 1.062 | 0.50 |
| Total Cholesterol (mg/Dl) | 1.084 | -0.019 | 0.045 | 0.982 | 0.899 | 1.071 | 0.68 |
| Total Cholesterol (mg/Dl) | 1.399 | -0.003 | 0.043 | 0.997 | 0.915 | 1.085 | 0.94 |
| Total Cholesterol (mg/Dl) | 1.736 | 0.018 | 0.043 | 1.018 | 0.936 | 1.108 | 0.67 |
| Total Cholesterol (mg/Dl) | 2.160 | 0.022 | 0.043 | 1.022 | 0.939 | 1.112 | 0.61 |
| Total Cholesterol (mg/Dl) | 2.677 | 0.01 | 0.044 | 1.010 | 0.926 | 1.102 | 0.82 |
| Total Cholesterol (mg/Dl) | 3.294 | -0.002 | 0.047 | 0.998 | 0.91 | 1.095 | 0.97 |
| Total Cholesterol (mg/Dl) | 3.962 | -0.019 | 0.051 | 0.982 | 0.887 | 1.086 | 0.72 |
| Total Cholesterol (mg/Dl) | 4.750 | -0.03 | 0.057 | 0.971 | 0.869 | 1.085 | 0.60 |
|  |  |  |  |  |  |  |  |
| Residual Urine output (%) | 0.003 | -0.318 | 0.185 | 0.727 | 0.506 | 1.046 | 0.09 |
| Residual Urine output (%) | 0.444 | -0.315 | 0.168 | 0.730 | 0.525 | 1.014 | 0.06 |
| Residual Urine output (%) | 0.794 | -0.206 | 0.152 | 0.813 | 0.603 | 1.097 | 0.18 |
| Residual Urine output (%) | 1.084 | -0.068 | 0.143 | 0.934 | 0.706 | 1.236 | 0.63 |
| Residual Urine output (%) | 1.399 | -0.017 | 0.138 | 0.984 | 0.75 | 1.289 | 0.91 |
| Residual Urine output (%) | 1.736 | 0.089 | 0.137 | 1.093 | 0.835 | 1.431 | 0.52 |
| Residual Urine output (%) | 2.160 | 0.053 | 0.14 | 1.055 | 0.802 | 1.387 | 0.70 |
| Residual Urine output (%) | 2.677 | 0.116 | 0.147 | 1.123 | 0.842 | 1.498 | 0.43 |
| Residual Urine output (%) | 3.294 | 0.178 | 0.159 | 1.195 | 0.875 | 1.634 | 0.26 |
| Residual Urine output (%) | 3.962 | 0.195 | 0.176 | 1.215 | 0.86 | 1.716 | 0.27 |
| Residual Urine output (%) | 4.750 | 0.18 | 0.192 | 1.198 | 0.822 | 1.744 | 0.35 |
| ^$^LL=Lower limit of 95% CI of the HR estimate; UL=Upper limit of 95% CI of the HR estimate; | | | | | | | |
