# Supplementary material for: All-cause and cause-specific mortality associated with diabetes in prevalent hemodialysis patients
Source: BMC Nephrol. 2012 Oct 1;13:130. doi: 10.1186/1471-2369-13-130 (PMC3519533; doi:10.1186/1471-2369-13-130)
Supplement: Additional file 2 — Table S3. Detail HR estimates from the Gray’s model for all time intervals. [file 1471-2369-13-130-S2.doc]

**Supplementary table**

Table S3: Detail HR estimates from the Gray's model for all time intervals

| **Variables** | **Time** | **Coeff** | **Std Err** | **HR** | **^$^LL** | **^$^UL** | **p-value** |
| --- | --- | --- | --- | --- | --- | --- | --- |
| High Kt/V | 0.279 | 0.041 | 0.119 | 1.042 | 0.825 | 1.317 | 0.73 |
| High Kt/V | 0.832 | 0.024 | 0.108 | 1.024 | 0.828 | 1.267 | 0.83 |
| High Kt/V | 1.079 | 0.017 | 0.099 | 1.017 | 0.837 | 1.235 | 0.87 |
| High Kt/V | 1.352 | -0.036 | 0.093 | 0.965 | 0.803 | 1.159 | 0.70 |
| High Kt/V | 1.659 | -0.015 | 0.091 | 0.985 | 0.825 | 1.176 | 0.87 |
| High Kt/V | 1.985 | -0.024 | 0.09 | 0.976 | 0.819 | 1.164 | 0.79 |
| High Kt/V | 2.461 | -0.084 | 0.091 | 0.919 | 0.77 | 1.098 | 0.35 |
| High Kt/V | 2.888 | -0.065 | 0.094 | 0.937 | 0.78 | 1.126 | 0.49 |
| High Kt/V | 3.488 | -0.075 | 0.099 | 0.928 | 0.763 | 1.128 | 0.45 |
| High Kt/V | 4.057 | -0.106 | 0.109 | 0.9 | 0.727 | 1.113 | 0.33 |
| High Kt/V | 4.821 | -0.114 | 0.12 | 0.893 | 0.706 | 1.129 | 0.34 |
| High Flux | 0.279 | -0.187 | 0.12 | 0.829 | 0.656 | 1.048 | 0.12 |
| High Flux | 0.832 | -0.166 | 0.109 | 0.847 | 0.685 | 1.048 | 0.13 |
| High Flux | 1.079 | -0.14 | 0.099 | 0.869 | 0.715 | 1.056 | 0.16 |
| High Flux | 1.352 | -0.143 | 0.094 | 0.867 | 0.722 | 1.041 | 0.13 |
| High Flux | 1.659 | -0.13 | 0.091 | 0.878 | 0.735 | 1.049 | 0.15 |
| High Flux | 1.985 | -0.125 | 0.09 | 0.882 | 0.74 | 1.052 | 0.16 |
| High Flux | 2.461 | -0.078 | 0.091 | 0.925 | 0.774 | 1.106 | 0.39 |
| High Flux | 2.888 | -0.013 | 0.094 | 0.987 | 0.821 | 1.187 | 0.89 |
| High Flux | 3.488 | 0.093 | 0.1 | 1.098 | 0.902 | 1.335 | 0.35 |
| High Flux | 4.057 | 0.15 | 0.109 | 1.162 | 0.938 | 1.44 | 0.17 |
| High Flux | 4.821 | 0.205 | 0.12 | 1.228 | 0.97 | 1.554 | 0.09 |
| Age | 0.279 | 0.346 | 0.055 | 1.413 | 1.268 | 1.575 | 0.00 |
| Age | 0.832 | 0.357 | 0.051 | 1.43 | 1.295 | 1.579 | 0.00 |
| Age | 1.079 | 0.345 | 0.047 | 1.411 | 1.288 | 1.547 | 0.00 |
| Age | 1.352 | 0.364 | 0.044 | 1.439 | 1.32 | 1.57 | 0.00 |
| Age | 1.659 | 0.362 | 0.043 | 1.436 | 1.32 | 1.562 | 0.00 |
| Age | 1.985 | 0.348 | 0.043 | 1.416 | 1.303 | 1.539 | 0.00 |
| Age | 2.461 | 0.365 | 0.043 | 1.441 | 1.324 | 1.567 | 0.00 |
| Age | 2.888 | 0.393 | 0.044 | 1.481 | 1.357 | 1.615 | 0.00 |
| Age | 3.488 | 0.385 | 0.047 | 1.47 | 1.341 | 1.612 | 0.00 |
| Age | 4.057 | 0.379 | 0.051 | 1.46 | 1.321 | 1.614 | 0.00 |
| Age | 4.821 | 0.393 | 0.056 | 1.481 | 1.328 | 1.652 | 0.00 |
| Black Race | 0.279 | -0.356 | 0.125 | 0.701 | 0.548 | 0.895 | 0.00 |
| Black Race | 0.832 | -0.381 | 0.113 | 0.683 | 0.547 | 0.854 | 0.00 |
| Black Race | 1.079 | -0.316 | 0.104 | 0.729 | 0.595 | 0.894 | 0.00 |
| Black Race | 1.352 | -0.301 | 0.098 | 0.74 | 0.611 | 0.897 | 0.00 |
| Black Race | 1.659 | -0.373 | 0.096 | 0.688 | 0.571 | 0.83 | 0.00 |
| Black Race | 1.985 | -0.404 | 0.095 | 0.668 | 0.554 | 0.805 | 0.00 |
| Black Race | 2.461 | -0.423 | 0.097 | 0.655 | 0.542 | 0.792 | 0.00 |
| Black Race | 2.888 | -0.427 | 0.102 | 0.653 | 0.535 | 0.796 | 0.00 |
| Black Race | 3.488 | -0.32 | 0.109 | 0.726 | 0.587 | 0.899 | 0.00 |
| Black Race | 4.057 | -0.231 | 0.12 | 0.794 | 0.628 | 1.004 | 0.05 |
| Black Race | 4.821 | -0.187 | 0.131 | 0.829 | 0.641 | 1.072 | 0.15 |
| BMI | 0.279 | -0.022 | 0.013 | 0.978 | 0.954 | 1.003 | 0.08 |
| BMI | 0.832 | -0.022 | 0.012 | 0.979 | 0.957 | 1.001 | 0.06 |
| BMI | 1.079 | -0.015 | 0.011 | 0.985 | 0.965 | 1.006 | 0.15 |
| BMI | 1.352 | -0.013 | 0.01 | 0.987 | 0.968 | 1.007 | 0.19 |
| BMI | 1.659 | -0.018 | 0.01 | 0.983 | 0.964 | 1.002 | 0.07 |
| BMI | 1.985 | -0.028 | 0.01 | 0.973 | 0.954 | 0.991 | 0.00 |
| BMI | 2.461 | -0.036 | 0.01 | 0.965 | 0.946 | 0.983 | 0.00 |
| BMI | 2.888 | -0.046 | 0.01 | 0.955 | 0.936 | 0.974 | 0.00 |
| BMI | 3.488 | -0.05 | 0.011 | 0.951 | 0.931 | 0.972 | 0.00 |
| BMI | 4.057 | -0.054 | 0.012 | 0.948 | 0.926 | 0.97 | 0.00 |
| BMI | 4.821 | -0.049 | 0.013 | 0.952 | 0.928 | 0.976 | 0.00 |
| Duration | 0.279 | 0.035 | 0.014 | 1.035 | 1.007 | 1.064 | 0.01 |
| Duration | 0.832 | 0.028 | 0.013 | 1.028 | 1.003 | 1.054 | 0.03 |
| Duration | 1.079 | 0.028 | 0.012 | 1.028 | 1.005 | 1.052 | 0.02 |
| Duration | 1.352 | 0.025 | 0.011 | 1.025 | 1.003 | 1.048 | 0.03 |
| Duration | 1.659 | 0.023 | 0.011 | 1.023 | 1.001 | 1.045 | 0.04 |
| Duration | 1.985 | 0.024 | 0.011 | 1.024 | 1.002 | 1.046 | 0.03 |
| Duration | 2.461 | 0.03 | 0.011 | 1.03 | 1.008 | 1.053 | 0.01 |
| Duration | 2.888 | 0.031 | 0.012 | 1.031 | 1.008 | 1.055 | 0.01 |
| Duration | 3.488 | 0.025 | 0.012 | 1.025 | 1.001 | 1.05 | 0.05 |
| Duration | 4.057 | 0.02 | 0.013 | 1.02 | 0.993 | 1.047 | 0.14 |
| Duration | 4.821 | 0.01 | 0.015 | 1.01 | 0.981 | 1.04 | 0.50 |
| Access(Catheter vs. All other) | 0.279 | 0.548 | 0.223 | 1.729 | 1.117 | 2.677 | 0.01 |
| Access(Catheter vs. All other) | 0.832 | 0.424 | 0.2 | 1.528 | 1.033 | 2.26 | 0.03 |
| Access(Catheter vs. All other) | 1.079 | 0.344 | 0.185 | 1.41 | 0.981 | 2.027 | 0.06 |
| Access(Catheter vs. All other) | 1.352 | 0.41 | 0.18 | 1.506 | 1.058 | 2.144 | 0.02 |
| Access(Catheter vs. All other) | 1.659 | 0.447 | 0.18 | 1.563 | 1.099 | 2.224 | 0.01 |
| Access(Catheter vs. All other) | 1.985 | 0.546 | 0.185 | 1.726 | 1.2 | 2.482 | 0.00 |
| Access(Catheter vs. All other) | 2.461 | 0.599 | 0.195 | 1.82 | 1.241 | 2.67 | 0.00 |
| Access(Catheter vs. All other) | 2.888 | 0.619 | 0.212 | 1.856 | 1.225 | 2.814 | 0.00 |
| Access(Catheter vs. All other) | 3.488 | 0.69 | 0.233 | 1.995 | 1.264 | 3.148 | 0.00 |
| Access(Catheter vs. All other) | 4.057 | 0.548 | 0.255 | 1.73 | 1.049 | 2.855 | 0.03 |
| Access(Catheter vs. All other) | 4.821 | 0.509 | 0.275 | 1.663 | 0.97 | 2.852 | 0.07 |
| Comorbidity ICED Score | 0.279 | 0.208 | 0.077 | 1.231 | 1.06 | 1.43 | 0.01 |
| Comorbidity ICED Score | 0.832 | 0.253 | 0.07 | 1.288 | 1.124 | 1.476 | 0.00 |
| Comorbidity ICED Score | 1.079 | 0.273 | 0.064 | 1.314 | 1.16 | 1.489 | 0.00 |
| Comorbidity ICED Score | 1.352 | 0.27 | 0.06 | 1.31 | 1.164 | 1.473 | 0.00 |
| Comorbidity ICED Score | 1.659 | 0.277 | 0.058 | 1.319 | 1.176 | 1.478 | 0.00 |
| Comorbidity ICED Score | 1.985 | 0.314 | 0.058 | 1.369 | 1.223 | 1.532 | 0.00 |
| Comorbidity ICED Score | 2.461 | 0.344 | 0.058 | 1.411 | 1.259 | 1.581 | 0.00 |
| Comorbidity ICED Score | 2.888 | 0.369 | 0.06 | 1.446 | 1.285 | 1.627 | 0.00 |
| Comorbidity ICED Score | 3.488 | 0.381 | 0.064 | 1.464 | 1.292 | 1.659 | 0.00 |
| Comorbidity ICED Score | 4.057 | 0.419 | 0.07 | 1.521 | 1.327 | 1.743 | 0.00 |
| Comorbidity ICED Score | 4.821 | 0.435 | 0.076 | 1.546 | 1.331 | 1.795 | 0.00 |
| Blood Pressure | 0.279 | -0.12 | 0.073 | 0.887 | 0.768 | 1.024 | 0.10 |
| Systolic | 0.832 | -0.127 | 0.068 | 0.881 | 0.771 | 1.006 | 0.06 |
| Systolic | 1.079 | -0.122 | 0.063 | 0.885 | 0.782 | 1.001 | 0.05 |
| Systolic | 1.352 | -0.115 | 0.06 | 0.892 | 0.793 | 1.003 | 0.06 |
| Systolic | 1.659 | -0.075 | 0.058 | 0.928 | 0.828 | 1.041 | 0.20 |
| Systolic | 1.985 | 0 | 0.058 | 1 | 0.893 | 1.12 | 1.00 |
| Systolic | 2.461 | 0.062 | 0.058 | 1.064 | 0.949 | 1.193 | 0.29 |
| Systolic | 2.888 | 0.077 | 0.06 | 1.08 | 0.961 | 1.215 | 0.20 |
| Systolic | 3.488 | 0.047 | 0.063 | 1.048 | 0.927 | 1.184 | 0.46 |
| Systolic | 4.057 | 0.055 | 0.067 | 1.056 | 0.927 | 1.204 | 0.41 |
| Systolic | 4.821 | 0.064 | 0.072 | 1.066 | 0.925 | 1.227 | 0.38 |
| Diastolic | 0.279 | -0.052 | 0.078 | 0.949 | 0.815 | 1.105 | 0.50 |
| Diastolic | 0.832 | -0.087 | 0.072 | 0.917 | 0.797 | 1.055 | 0.23 |
| Diastolic | 1.079 | -0.074 | 0.067 | 0.929 | 0.815 | 1.058 | 0.27 |
| Diastolic | 1.352 | -0.103 | 0.063 | 0.902 | 0.797 | 1.022 | 0.11 |
| Diastolic | 1.659 | -0.112 | 0.061 | 0.894 | 0.793 | 1.009 | 0.07 |
| Diastolic | 1.985 | -0.099 | 0.061 | 0.905 | 0.804 | 1.02 | 0.10 |
| Diastolic | 2.461 | -0.103 | 0.062 | 0.902 | 0.8 | 1.018 | 0.09 |
| Diastolic | 2.888 | -0.106 | 0.064 | 0.899 | 0.794 | 1.019 | 0.10 |
| Diastolic | 3.488 | -0.073 | 0.067 | 0.93 | 0.815 | 1.061 | 0.28 |
| Diastolic | 4.057 | -0.053 | 0.073 | 0.949 | 0.823 | 1.094 | 0.47 |
| Diastolic | 4.821 | -0.058 | 0.078 | 0.944 | 0.81 | 1.1 | 0.46 |
| Smoking (Smoked vs. Never) | 0.279 | 0.201 | 0.083 | 1.223 | 1.039 | 1.438 | 0.02 |
| Smoking (Smoked vs. Never) | 0.832 | 0.217 | 0.075 | 1.242 | 1.072 | 1.44 | 0.00 |
| Smoking (Smoked vs. Never) | 1.079 | 0.207 | 0.069 | 1.23 | 1.074 | 1.408 | 0.00 |
| Smoking (Smoked vs. Never) | 1.352 | 0.194 | 0.065 | 1.214 | 1.068 | 1.379 | 0.00 |
| Smoking (Smoked vs. Never) | 1.659 | 0.174 | 0.063 | 1.191 | 1.052 | 1.347 | 0.01 |
| Smoking (Smoked vs. Never) | 1.985 | 0.156 | 0.063 | 1.168 | 1.033 | 1.321 | 0.01 |
| Smoking (Smoked vs. Never) | 2.461 | 0.112 | 0.064 | 1.118 | 0.987 | 1.266 | 0.08 |
| Smoking (Smoked vs. Never) | 2.888 | 0.104 | 0.066 | 1.11 | 0.976 | 1.263 | 0.11 |
| Smoking (Smoked vs. Never) | 3.488 | 0.055 | 0.07 | 1.056 | 0.921 | 1.211 | 0.44 |
| Smoking (Smoked vs. Never) | 4.057 | 0.047 | 0.076 | 1.048 | 0.902 | 1.217 | 0.54 |
| Smoking (Smoked vs. Never) | 4.821 | 0.028 | 0.084 | 1.029 | 0.873 | 1.212 | 0.74 |
| Calcium (mg/dL) | 0.279 | -0.112 | 0.06 | 0.894 | 0.795 | 1.005 | 0.06 |
| Calcium (mg/dL) | 0.832 | -0.112 | 0.054 | 0.894 | 0.803 | 0.995 | 0.04 |
| Calcium (mg/dL) | 1.079 | -0.121 | 0.05 | 0.886 | 0.804 | 0.977 | 0.02 |
| Calcium (mg/dL) | 1.352 | -0.138 | 0.047 | 0.871 | 0.794 | 0.955 | 0.00 |
| Calcium (mg/dL) | 1.659 | -0.1 | 0.046 | 0.905 | 0.828 | 0.99 | 0.03 |
| Calcium (mg/dL) | 1.985 | -0.047 | 0.045 | 0.954 | 0.874 | 1.042 | 0.30 |
| Calcium (mg/dL) | 2.461 | 0.001 | 0.045 | 1.001 | 0.917 | 1.094 | 0.97 |
| Calcium (mg/dL) | 2.888 | -0.008 | 0.046 | 0.992 | 0.906 | 1.086 | 0.86 |
| Calcium (mg/dL) | 3.488 | -0.022 | 0.049 | 0.979 | 0.889 | 1.077 | 0.66 |
| Calcium (mg/dL) | 4.057 | -0.033 | 0.053 | 0.967 | 0.871 | 1.074 | 0.53 |
| Calcium (mg/dL) | 4.821 | -0.058 | 0.059 | 0.944 | 0.841 | 1.059 | 0.32 |
| Phosphorus (mg/dL) | 0.279 | 0.125 | 0.061 | 1.133 | 1.005 | 1.279 | 0.04 |
| Phosphorus (mg/dL) | 0.832 | 0.127 | 0.056 | 1.135 | 1.018 | 1.267 | 0.02 |
| Phosphorus (mg/dL) | 1.079 | 0.129 | 0.051 | 1.138 | 1.029 | 1.258 | 0.01 |
| Phosphorus (mg/dL) | 1.352 | 0.141 | 0.049 | 1.152 | 1.047 | 1.267 | 0.00 |
| Phosphorus (mg/dL) | 1.659 | 0.112 | 0.047 | 1.118 | 1.019 | 1.227 | 0.02 |
| Phosphorus (mg/dL) | 1.985 | 0.086 | 0.047 | 1.09 | 0.993 | 1.195 | 0.07 |
| Phosphorus (mg/dL) | 2.461 | 0.085 | 0.048 | 1.088 | 0.991 | 1.195 | 0.08 |
| Phosphorus (mg/dL) | 2.888 | 0.098 | 0.05 | 1.103 | 1 | 1.216 | 0.05 |
| Phosphorus (mg/dL) | 3.488 | 0.111 | 0.053 | 1.118 | 1.007 | 1.24 | 0.04 |
| Phosphorus (mg/dL) | 4.057 | 0.109 | 0.058 | 1.115 | 0.995 | 1.249 | 0.06 |
| Phosphorus (mg/dL) | 4.821 | 0.107 | 0.063 | 1.113 | 0.983 | 1.261 | 0.09 |
| Albumin(mg/dL) | 0.279 | -0.489 | 0.093 | 0.613 | 0.511 | 0.736 | 0.00 |
| Albumin(mg/dL) | 0.832 | -0.463 | 0.084 | 0.629 | 0.534 | 0.743 | 0.00 |
| Albumin(mg/dL) | 1.079 | -0.466 | 0.077 | 0.628 | 0.539 | 0.73 | 0.00 |
| Albumin(mg/dL) | 1.352 | -0.455 | 0.073 | 0.635 | 0.55 | 0.733 | 0.00 |
| Albumin(mg/dL) | 1.659 | -0.387 | 0.071 | 0.679 | 0.59 | 0.781 | 0.00 |
| Albumin(mg/dL) | 1.985 | -0.349 | 0.071 | 0.706 | 0.614 | 0.811 | 0.00 |
| Albumin(mg/dL) | 2.461 | -0.274 | 0.072 | 0.761 | 0.66 | 0.876 | 0.00 |
| Albumin(mg/dL) | 2.888 | -0.259 | 0.075 | 0.772 | 0.667 | 0.894 | 0.00 |
| Albumin(mg/dL) | 3.488 | -0.288 | 0.08 | 0.75 | 0.641 | 0.877 | 0.00 |
| Albumin(mg/dL) | 4.057 | -0.307 | 0.087 | 0.736 | 0.62 | 0.873 | 0.00 |
| Albumin(mg/dL) | 4.821 | -0.313 | 0.096 | 0.731 | 0.606 | 0.882 | 0.00 |
| Diabetes | 0.279 | 0.347 | 0.163 | 1.415 | 1.028 | 1.947 | 0.03 |
| Diabetes | 0.832 | 0.385 | 0.151 | 1.47 | 1.094 | 1.977 | 0.01 |
| Diabetes | 1.079 | 0.343 | 0.142 | 1.41 | 1.067 | 1.862 | 0.02 |
| Diabetes | 1.352 | 0.359 | 0.136 | 1.432 | 1.097 | 1.871 | 0.01 |
| Diabetes | 1.659 | 0.401 | 0.133 | 1.494 | 1.15 | 1.94 | 0.00 |
| Diabetes | 1.985 | 0.445 | 0.133 | 1.56 | 1.202 | 2.024 | 0.00 |
| Diabetes | 2.461 | 0.384 | 0.135 | 1.468 | 1.128 | 1.912 | 0.00 |
| Diabetes | 2.888 | 0.432 | 0.139 | 1.541 | 1.173 | 2.023 | 0.00 |
| Diabetes | 3.488 | 0.534 | 0.146 | 1.706 | 1.282 | 2.27 | 0.00 |
| Diabetes | 4.057 | 0.686 | 0.155 | 1.985 | 1.464 | 2.692 | 0.00 |
| Diabetes | 4.821 | 0.792 | 0.166 | 2.208 | 1.594 | 3.059 | 0.00 |
| Female Sex | 0.279 | -0.009 | 0.156 | 0.991 | 0.731 | 1.345 | 0.96 |
| Female Sex | 0.832 | 0 | 0.144 | 1 | 0.754 | 1.327 | 1.00 |
| Female Sex | 1.079 | 0.046 | 0.135 | 1.047 | 0.804 | 1.364 | 0.74 |
| Female Sex | 1.352 | 0.019 | 0.129 | 1.019 | 0.792 | 1.313 | 0.88 |
| Female Sex | 1.659 | 0.043 | 0.126 | 1.043 | 0.816 | 1.335 | 0.74 |
| Female Sex | 1.985 | 0.063 | 0.125 | 1.065 | 0.834 | 1.359 | 0.62 |
| Female Sex | 2.461 | 0.072 | 0.126 | 1.074 | 0.839 | 1.375 | 0.57 |
| Female Sex | 2.888 | 0.092 | 0.13 | 1.097 | 0.851 | 1.414 | 0.48 |
| Female Sex | 3.488 | 0.117 | 0.136 | 1.124 | 0.861 | 1.467 | 0.39 |
| Female Sex | 4.057 | 0.172 | 0.145 | 1.187 | 0.893 | 1.579 | 0.24 |
| Female Sex | 4.821 | 0.203 | 0.157 | 1.225 | 0.902 | 1.665 | 0.19 |
| Diabetes*Sex | 0.279 | -0.176 | 0.202 | 0.839 | 0.565 | 1.245 | 0.38 |
| Diabetes*Sex | 0.832 | -0.161 | 0.189 | 0.851 | 0.587 | 1.233 | 0.39 |
| Diabetes*Sex | 1.079 | -0.211 | 0.18 | 0.81 | 0.569 | 1.151 | 0.24 |
| Diabetes*Sex | 1.352 | -0.245 | 0.173 | 0.782 | 0.557 | 1.099 | 0.16 |
| Diabetes*Sex | 1.659 | -0.282 | 0.17 | 0.754 | 0.541 | 1.052 | 0.10 |
| Diabetes*Sex | 1.985 | -0.268 | 0.169 | 0.765 | 0.55 | 1.065 | 0.11 |
| Diabetes*Sex | 2.461 | -0.34 | 0.171 | 0.712 | 0.509 | 0.994 | 0.05 |
| Diabetes*Sex | 2.888 | -0.45 | 0.175 | 0.638 | 0.453 | 0.899 | 0.01 |
| Diabetes*Sex | 3.488 | -0.525 | 0.181 | 0.592 | 0.415 | 0.844 | 0.00 |
| Diabetes*Sex | 4.057 | -0.396 | 0.19 | 0.673 | 0.463 | 0.977 | 0.04 |
| Diabetes*Sex | 4.821 | -0.331 | 0.202 | 0.718 | 0.483 | 1.067 | 0.10 |
| ^$^LL=Lower limit of 95% CI of the HR estimate; UL=Upper limit of 95% CI of the HR estimate; | | | | | | | |
